# Different effects of metformin and insulin on primary and secondary chemoprevention of colorectal adenoma in diabetes type 2: Traditional and Bayesian meta-analysis

**DOI:** 10.17179/excli2017-993

**Published:** 2018-01-08

**Authors:** Marjan Mansourian, Raheleh Karimi, Golnaz Vaseghi

**Affiliations:** 1 Epidemiology and Biostatistics Department, School of Health, Isfahan University of Medical Sciences, Isfahan, Iran; 2 Student Research Committee, School of Health, Isfahan University of Medical Sciences, Isfahan, Iran; 3 Isfahan Cardiovascular Research Center, Cardiovascular Research Institute, Isfahan University of Medical Sciences, Isfahan, Iran

**Keywords:** metformin, insulin, adenoma, colonic polyps, diabetes mellitus type 2, meta-analysis

## Abstract

Metformin and insulin differently affect the risk of colon cancer in type 2 diabetic patients, however their effects on colon adenoma is not clear. PubMed, ISI, Scopus and Cochrane databases were searched for studies reporting. The outcomes were total adenoma; advanced adenoma and recurrent adenoma. Traditional and Bayesian meta-analysis were conducted via random-effects models. Odds ratios (OR) with 95 % confidence intervals (CIs) / or credible intervals (CrI) were used to describe the ratio of different events. A random-effects model described by DerSimonian and Laird was performed, when significant between-study heterogeneity existed. Alternatively, an inverse variance fixed effects model was used, when there was no significant heterogeneity across studies. The potential publication bias was assessed with funnel plot, Egger and Begg's regression asymmetry tests. Moreover, “trim and fill” procedure was used to assess the possible effect of publication bias. For metformin intake, 11 studies (51991 patients) were included. The results showed that metformin significantly decreased the risk of advance adenoma (OR= 0.51, p< 0.001). The risk of total adenoma was not associated with metformin use (OR= 0.86, p=0.274), and metformin did not affect the risk of adenoma recurrence (OR= 0.89, p=0.137). Five studies (2678 patients) were included in the analysis to determine the effect of insulin therapy. Insulin significantly increased the risk of colorectal adenoma (OR= 1.43, p=0.002). These findings indicate that metformin has no protective effect on total and recurrent adenoma, whilst it significantly reduces the risk of advanced adenoma, but insulin increases the risk of total adenoma.

## Abbreviations

CRC: Colorectal cancer

T2DM: Type 2 diabetes mellitus

AMPK: AMP-activated protein kinase

CI: Confidence interval

CrI: Credibility interval

OR: Odds ratio

MCMC: Markov chain Monte Carlo

## Introduction

Colorectal cancer (CRC) is one of the most common cancer globally, it has been shown that the incidence of CRC has been increasing worldwide (Torre et al., 2015[36]). Colorectal adenomas are relatively prone to develop into colorectal carcinoma (Morson, 1974[30]). Accordingly, therapies that lessen adenoma recurrence may additionally lessen both CRC risk and the necessity for colonoscopy (Lieberman et al., 2005[27]). One of the well-known risk factor for CRC is type 2 diabetes; hyperinsulinemia caused by insulin resistance, influences the incidence of CRC (Weiderpass et al., 1997[38]). However, exogenous insulin leads to higher concentration of systemic hyper-insulinemia state compare to endogenous hyper-insulinemia (Genuth, 1990[15]). Recent studies have reported a significant increase in risk of CRC and colorectal adenomas, respectively, among patients with type 2 diabetes on insulin therapy compared to non-insulin users (Chung et al., 2008[7]; Yang et al., 2004[40]), but the data are still conflicting.

Metformin is another potent anti-hyperglycemic drug. It can decrease hyperinsulinemia, increase insulin sensitivity (Ashokkumar et al., 2006[1]) and reduce blood glucose concentrations (Viollet et al., 2012[37]). Metformin also activates liver kinase B1-dependent AMP-activated protein kinase (AMPK) in the liver, activated AMPK inhibits the proliferation and growth of cells by inhibiting mammalian target of rapamycin (Sarbassov et al., 2005[31]). 

Some animal studies have revealed that metformin prevents the proliferation of colonic epithelial cells (Tomimoto et al., 2008[35]). A few scientists showed that metformin might be a potential defensive factor of colorectal adenomas and colorectal disease in T2DM patients (Cho et al., 2014[6]; Chung et al., 2008[7]; Jain et al., 2016[18]; Kanadiya et al., 2013[20]; Kim et al., 2015[22]). However, some different investigations found no connection between metformin treatment and colorectal adenomas or colorectal malignancy (Eddi et al., 2012[12]; Lewis et al., 2007[26]), thus the results were still inconsistent. Inconsistencies in these results might cause by variations in study style, populations, or totally different applied mathematics strategies. Some researchers have performed meta-analysis to assess the link between metformin use and adenoma risk (Jung et al., 2017[19]). It has been suggested that CRC risk among diabetes may vary with the type of treatment (Eddi et al., 2012[12]). So we conduct this study to explore associations between metformin or insulin use and adenomas risk, the impact of metformin on prevention of secondary adenoma, and to investigate if different treatments affect the risk of adenoma.

## Material and Methods

### Search strategy 

A systematic search of the online databases PubMed, ISI, Scopus, and Cochrane Collaboration up to January 2017 without any restrictions was conducted to find published articles. Search terms were: (“Metformin”) OR (“Insulin”) AND (“Adenoma*” OR “Colorectal adenoma*” OR “Colon polyp*”OR “Colon adenoma *” OR “Colorectal polyps” OR “Advanced adenoma*”OR “Recurrent adenoma*” OR “Neoplasms”) AND (“Diabetes Mellitus, Type 2”OR“type 2diabetes”). All eligible studies were reviewed, and their bibliographies were checked for other relevant publications. For carrying out and reporting meta-analyses of observational studies, the standard criteria were followed. 

### Statistical analysis

Agreement on the selection of studies between the two reviewers was evaluated by the kappa coefficient. Meta-analysis was performed using both traditional and Bayesian meta-analysis. When the two methods produced different results, the results of Bayesian method was approved. The effect sizes were calculated based on total colorectal adenoma, advanced adenoma, and recurrence of adenoma in diabetic metformin users and also for the total colorectal adenomain diabetic insulin users compared with controls. Briefly, the odds ratio (OR, 95 % confidence intervals (CIs) / or credible intervals (CrI)) were used to describe the ratio of different events occurring in diabetic patients. An Inverse Variance fixed effects model was used to calculate the pooled effect measure. Otherwise, the random effects model described by DerSimonian and Laird (1986[10]) was applied if there was evidence of heterogeneity.

Heterogeneity between studies was assessed by I^2^ statistic and Cochrane's Q-statistic (Higgins et al., 2003[17]).

In the presence of significant statistical heterogeneity, apart from the random-effects model, sensitivity analyses were performed to evaluate the consistency of our results. Firstly, to evaluate any possible excessive influence of a single study, we examined whether the exclusion of this study substantially altered the magnitude or heterogeneity of the summary estimate. This was achieved by repeating the meta-analyses with exclusion of each individual study one at a time, to assess the overall effect of the exclusion on the pooled Ors (Sutton et al., 2000[33]).Secondly, subgroup analyses were performed by stratifying meta-analysis upon different factors that could potentially influence the results. These factors were established a priori to the analysis. We further explored heterogeneity by performing meta-regression analyses (method of moments) (Borenstein et al., 2009[5]).

In Bayesian hierarchical random-effects models (Best, 2005[4]; Sutton et al., 2000[34]), the first 10,000 iterations were discarded and results were reported as the posterior mean (OR) with 95 % (CrI) on the basis of a further 100,000 iterations. 

For the mean OR outcome, a normal prior with a large variance N(0,10^5^), as a vague prior, is placed upon the pooled effect size. As our meta-analysis include small numbers of studies, there was little information in the likelihood regarding the estimation of the between study variance parameter. In this situation. The prior can be influential in the analysis (Lambert et al., 2005[24]). In this regard, we defined Gamma distribution and Hulf-normal as an informative prior, in addition of using Uniform distribution for between study variance.

The likelihood of publication bias was assessed by constructing funnel plots (not shown), which were obtained by plotting the log ORs vs. SE of individual studies. Their symmetry was estimated by Egger's regression test and the Begg and Mazumdar adjusted rank correlation test (Begg and Mazumdar, 1994[3]; Copas and Shi, 2001[8]; Egger et al., 1997[13]). P values below 0.05 were interpreted as statistically significant, and the trim and fill method was used to further assess the possible effect of publication bias on the results of our meta-analysis (Duval and Tweedie, 2000[11]).The results of analysis was performed by Stata version 14 and Open BUGS version 3.2.3.

## Results

### Search results and study characteristics

**Figure 1:**
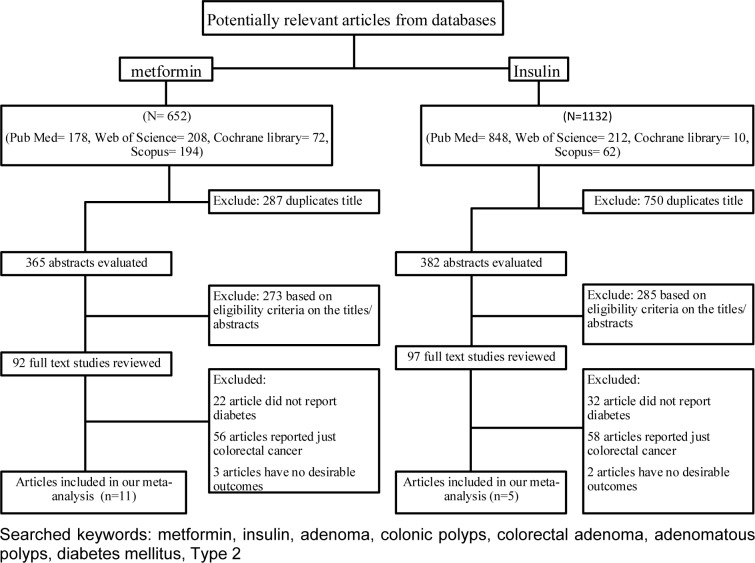
Flow chart of article selection process

A comprehensive literature review search, up to January 2017 was carried out (see Figure 1(Fig. 1)), overall, 652 potentially eligible studies were comforted to the inclusion criteria of this meta-analysis according metformin-treated diabetic patients. After excluding duplicates, we selected 365 records. Two hundred and eighty-seven studies were excluded because they did not meet the eligibility criteria based on their titles and abstracts. After reviewing the full texts, 11 studies were finally included. For studies containing insulin consumption, 1132 eligible studies were identified by keyword search. After excluding duplicates (N=750) and studies without eligibility criteria based on their titles, abstracts and full text (N=285), 5 studies were finally included.

The characteristics of each included studies and participants in association with metformin and insulin treatments are presented in Table 1(Tab. 1) (References in Table 1: Kim et al., 2015[22]; Kanadiya et al., 2013[20]; Cho et al., 2014[6]; Jain et al., 2016[18]; Eddi et al., 2012[12]; Lewis et al., 2007[26]; Chung et al., 2008[7]; Lee et al., 2012[25]; Marks et al., 2015[29]; Han et al., 2017[16]; Shin et al., 2013[32]) and 2(Tab. 2) (References in Table 2: Chung et al., 2008[7]; Jain et al., 2016[18]; Eddi et al., 2012[12]; Dash et al., 2013[9]; Wong et al., 2012[39]). Of the 11 studies presented based on metformin treatment (Table 1(Tab. 1)), 8 studies were retrospective cohort, 2 studies were case-control and only one study was a cross-sectional. In cohort studies, of the 51002 patients with type 2 diabetes, 5512 were assigned to the metformin group and 45490 of patients were assigned to non-metformin group. A total of 983 subjects in case-control studies, were randomly allocated to case (n=361) and control (n=622) groups. The mean age of patients who consumed metformin ranged from 60 to 71 years. Three studies reported both total and advanced adenoma, 4 studies were only about adenoma recurrence and 4 studies just reported total adenoma.

Of the 5 study presented for insulin use (Table 2(Tab. 2)), all studies were case-control and randomized assigned to case (n=1333) and control (n=1345) groups. The mean age of insulin recipient patients ranged from 54.6 to 71.

### Metformin therapy 

**Figure 2:**
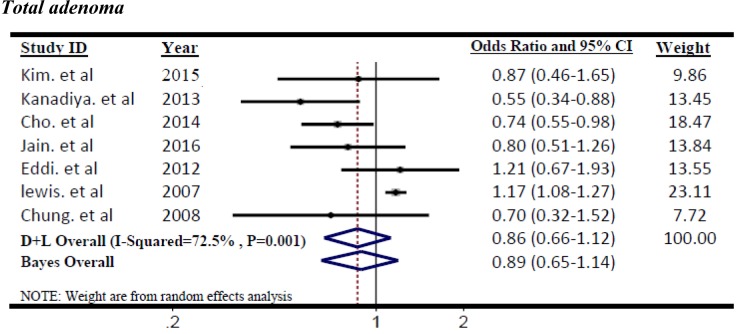
Forest plot of odds ratio of metformin on risk of Total adenoma, I^2^=72.5 %, Egger's test p-value=0.048

**Figure 3:**
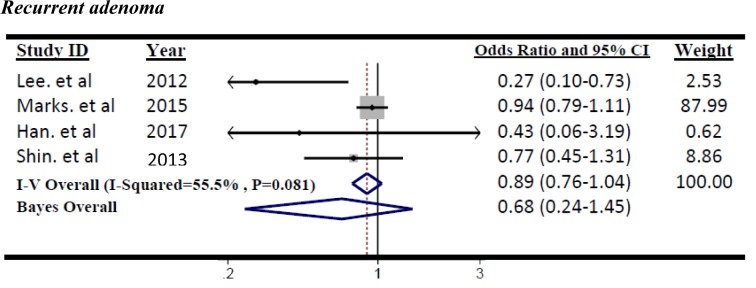
Forest plot of odds ratio of metformin on risk of Recurrent adenoma, I^2^=55.5 %, Egger's test p-value=0.152

Figure 2(Fig. 2) (References in Figure 2: Kim et al., 2015[22]; Kanadiya et al., 2013[20]; Cho et al., 2014[6]; Jain et al., 2016[18]; Eddi et al., 2012[12]; Lewis et al., 2007[26]; Chung et al., 2008[7]) shows the summary ORs of total adenoma incidence associated with metformin treatment for T2DM patients according to the 7 studies. Significant heterogeneity was found across the individual studies which enrolled in the meta-analysis (I^2^=72.5 %, p=0.001). Therefore, the effect size was pooled using the random-effects model. The metformin use was not significantly associated with reduced incidence of total adenoma among T2DM patients (OR=0.86, 95 % CI=0.66 to 1.12; p=0.274).The results of Bayesian hierarchical modeling were generally similar to those obtained using the traditional meta-analysis. The Bayesian credible intervals (CrI) were reasonably wider than the traditional confidence intervals (CI), (OR=0.89, 95 % CrI=0.65 to 1.14), as they accounted for additional variability. Also the risk of recurrence adenoma was not significantly lower among metformin users than controls (non-metformin users) pooled ORs (95 % CI) was 0.89 (0.76-1.04). Furthermore, the heterogeneity between 4 studies based on recurrent adenoma outcome was not significant (I^2^=55.5 %, p=0.08) then, the effect size was pooled using the fixed-effects model. (Figure 3(Fig. 3); References in Figure 3: Lee et al., 2012[25]; Marks et al., 2015[29]; Han et al., 2017[16]; Shin et al., 2013[32]).

**Figure 4:**
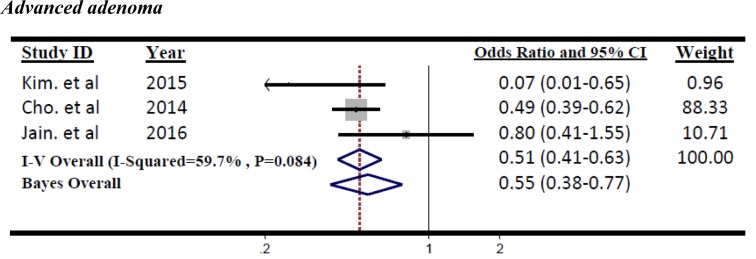
Forest plot of odds ratio of Metformin on risk of Advanced adenoma, I^2^=59.7 %, Egger's test p-value=0.816

The small number of studies on advanced adenoma leads to Bayesian meta-analysis. The significantly reduced advanced adenoma polyps was found in diabetes treated with metformin use (Figure 4(Fig. 4); References in Figure 4: Kim et al., 2015[22]; Cho et al., 2014[6]; Jain et al., 2016[18]), compared with other treatments according to Markov chain Monte Carlo (MCMC) Bayesian approach (OR=0.55, 95 % CrI=0.38 to 0.77). The non-significant heterogeneity was found between these three studies (I^2^=59.7 %, p=0.08). The result of both traditional and Bayesian approaches is shown in Table 3(Tab. 3).

For the Bayesian hierarchical modeling, we used several different prior distributions for parameters. The results showed that changing the form of supposed prior distribution had a minimal effect on pooled estimation. The results using N (0,10^5^) prior for mean OR and Gamma distribution for between study variance are presented in Table 3(Tab. 3).

Egger's linear regression test was applied to assess bias in the publication of three outcomes. The results revealed that there were no statistical evidence of publication bias using Egger's linear regression tests for advanced adenoma (P =0.816), and adenoma recurrence (P=0.152). For total adenoma, in order to quantify the amount of bias in the publication (P =0.048) the modification of the final meta-analysis results according to “trim and fill” method did not differ from the results of the Classic meta-analysis (summary estimate of OR (95 % CI)).

**Figure 5:**
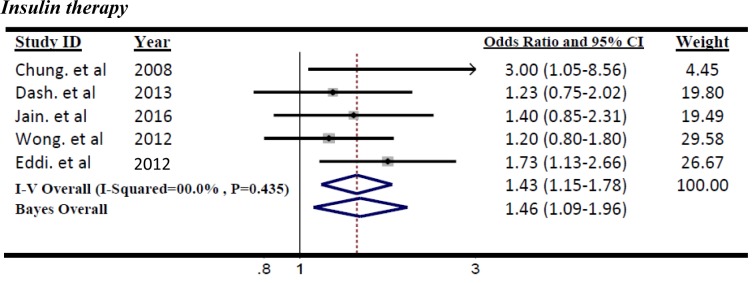
Forest plot of odds ratio of insulin therapy on risk of Colorectal adenoma, I^2^=0.0 %, Egger's test p-value=0. 207

The summary ORs of meta-analysis (OR=1.43, 95 % CI=1.15 to 1.78; P=0.002) revealed that there was a significant positive association between colorectal adenoma incidence and insulin intake between T2DM patients according to the 5 including studies (Figure 5(Fig. 5); References in Figure 5: Chung et al., 2008[7]; Dash et al., 2013[9]; Jain et al., 2016[18]; Wong et al, 2012[39]; Eddi et al., 2012[12]). Then the insulin recipient patients had a risk of colorectal adenoma compared with those who did not.

The significant heterogeneity was not identified (I^2^=0.0 %, p=0.435) in related meta-analysis. Therefore, the effect size pooled using the fixed-effects model. The results of Bayesian hierarchical modeling was inconsistent with the result of the traditional meta-analysis (OR=1.46, 95 % CrI=1.09 to 1.96) (Table 3(Tab. 3)). Also the result of the Egger's linear regression indicated no publication bias (P=0.207).

### Subgroup analysis

We conducted the subgroup analysis based on the study location, age of the patients and the study design (Table 4(Tab. 4)). The results of the subgroup analysis showed that in Korean studies, metformin therapy between T2DM patients significantly decreased the adenoma risk, totally. However, no significant relationship was found between studies from USA. This may be due to the dietary habits or lifestyle between these two areas. In an age-stratified subgroup analysis, the results demonstrated that the risk of total adenoma in patients whose age ≤ 65 years old in metformin group, was significantly lower than those whose age ≥ 65 years old (the annual risk of adenoma progression in patients whose age was ≥ 65 years old was 17 %). 

This may be for the age as a risk factor of colon polyps, especially in diabetic patients. The results of subgroup analyses based on the study design showed that there was no difference between cohort and case-control study design.

## Discussion

This meta-analysis sought to identify whether T2DM with and without metformin intake history have different risk in order to colorectal adenoma, using both conventional and Bayesian meta-analyses. Compared to traditional meta-analyses, we used also the Bayesian approach that takes into account all sources of variation and reflects these variations in the pooled result. The main results of this meta-analysis of 11 observational studies on the metformin use and risk of colorectal adenoma were three fold. First, analysis propose that metformin intake will significantly decrease the risk of advanced adenoma (OR= 0.51). Second, the risk of total adenoma is not associated with metformin use between type 2 diabetes (OR= 0.86) and third, results for the pooled ORs indicate that, there was no statistical association between metformin intake in diabetic patients and adenoma recurrence (OR= 0.89).In the subgroup analyses, after stratifying studies by location, we showed that metformin therapy was associated with a significant reduction in colorectal adenoma risk in Asian population, while no significant association was found in North Americans. This can be explained by dietary habits and/or other cultural and behavioural differences. For example, red meat and milk products, as well-known colorectal adenoma risks, are more popular in western countries (Aune et al., 2013[2]; Karagas et al., 1998[21]). Another subgroup analysis showed that in people younger than 65 years, metformin intake significantly reduced their risk. When studies were stratified by their designs no significant differences were observed. Previous studies have shown the protective effect of metformin on CRC (Liu et al., 2017[28]) and other cancers such as prostate and lung (Kourelis and Siegel, 2012[23]). It has been proposed that metformin can decrease the risk of colorectal adenoma by 24 %, but in our analysis we showed that metformin has no effect on primary adenoma risk which we believed in previous meta-analysis patients without diabetes have been included (Ford et al., 2012[14]) which may interfere with results. Metformin may have anti-cancer effects through direct (insulin-independent) and indirect (insulin-dependent) mechanisms. As insulin resistance, hyperinsulinemia and hyperglycemia are risk factors of cancers (Yang et al., 2004[40]), so the data in patients with diabetes should not merge with non-diabetes. However the efficacy of metformin in reducing advanced adenoma was similar to another analysis (Ford et al., 2012[14]). These results suggest that metformin may provide a greater protective effect against pre-neoplastic lesions than against adenoma itself. 

We also provided some evidence that metformin may not have beneficial effects against recurrence colorectal adenoma. The present study is an analysis based on the epidemiological studies which included large-scale and long-term follow-up results. Insulin significantly increases the risk of colorectal adenoma in T2DM patients (OR:1.43), however in this analysis due to lack of data we could not report data on stratified analysis by duration of insulin use duration of DM or age.

In conclusion, our meta-analysis indicates that metformin is associated with a reduction in risk of colorectal adenoma incidence in individuals with T2DM, compared with insulin treatment which significantly increases the risk of colon adenoma. So similar to CRC, DM medication may affect the risk of CRA.

## Acknowledgements

The authors would like to thank Dr. Erfan Sadeghi for his kindness. 

## Ethical approval

For this type of study, formal consent is not required.

## Conflict of interest

The authors declare that they have no conflict of interest.

## Funding

Master of sciences thesis in number of 395963.

## Figures and Tables

**Table 1:**
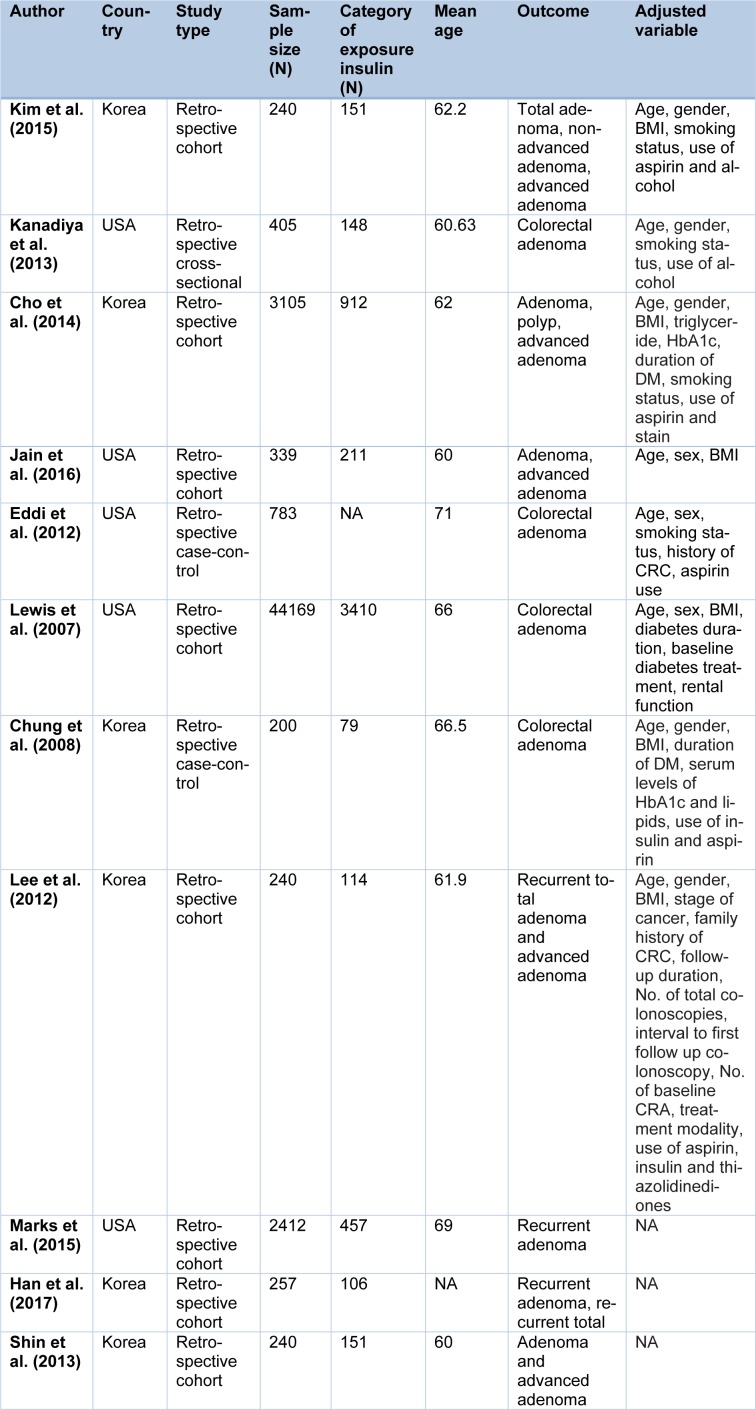
Characteristic of studies included in the meta-analysis (Metformin)

**Table 2:**
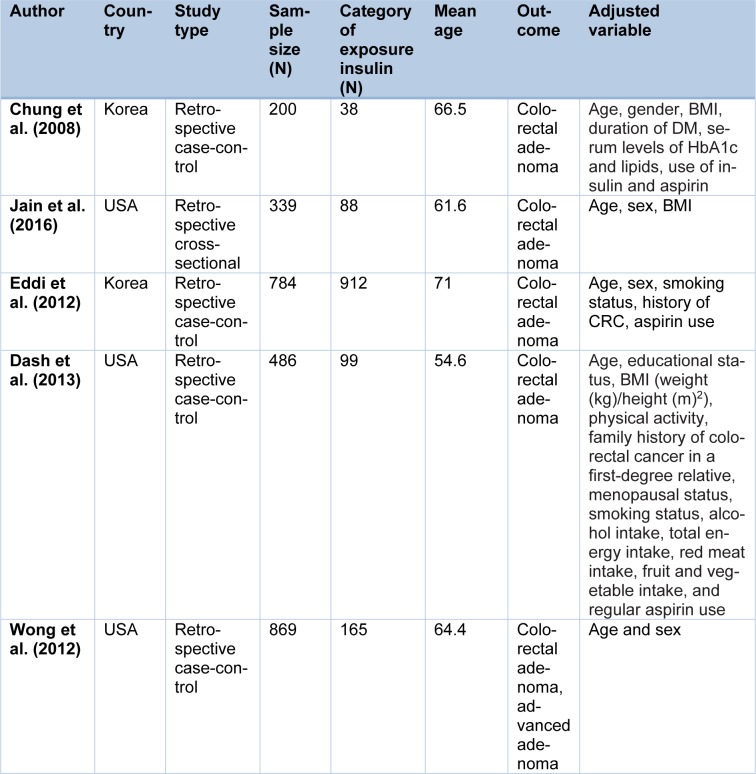
Characteristic of studies included in the meta-analysis (insulin)

**Table 3:**
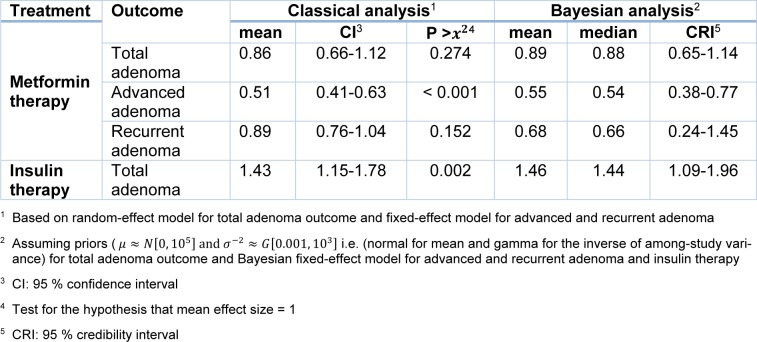
Mean effect sizes (OR) and corresponding statistics for a classical and Bayesian meta-analysis

**Table 4:**
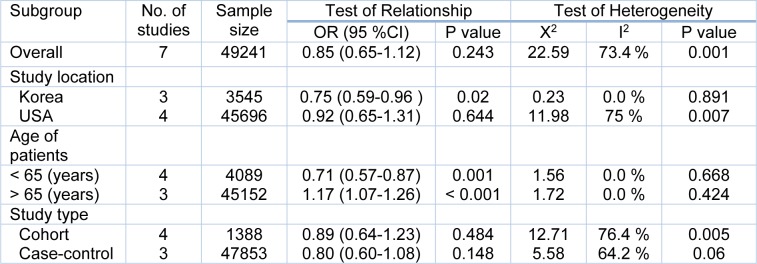
Subgroup analysis of relationship between metformin therapy and risk of total adenoma
